# Regional Anesthesia for Arthroscopic Knee Repair in a Patient With Hypertrophic Obstructive Cardiomyopathy (HOCM) Under Monitored Anesthesia Care With Dexmedetomidine Infusion

**DOI:** 10.7759/cureus.53862

**Published:** 2024-02-08

**Authors:** Julie J Cunningham, Andrew S Braun, Patrick Hussey, Amit Momaya, Promil Kukreja

**Affiliations:** 1 School of Medicine, University of Alabama at Birmingham (UAB), Birmingham, USA; 2 Anesthesiology and Perioperative Medicine, University of Alabama at Birmingham (UAB), Birmingham, USA; 3 Orthopedic Surgery, University of Alabama at Birmingham (UAB) School of Medicine, Birmingham, USA

**Keywords:** hypertrophic cardiomyopathy, monitored anesthesia care, peripheral nerve blockade, regional anesthesia, dexmedetomidine

## Abstract

Patients with hypertrophic obstructive cardiomyopathy (HOCM) who are scheduled for elective, noncardiac surgery present a distinctive challenge for perioperative healthcare providers. The use of general anesthesia and neuraxial anesthesia carries the risk of unpredictable hemodynamic changes and potential complications. Regional anesthesia (RA) emerges as a prudent and effective option for HOCM patients. RA provides advantages such as minimizing hemodynamic fluctuations, avoiding intubation, reducing pharmacologic side effects, facilitating enhanced recovery after surgery, and contributing to greater patient satisfaction. We share the case of a 15-year-old individual diagnosed with HOCM and exercise intolerance, undergoing arthroscopic repair for right patellar instability. In this instance, the patient received preoperative peripheral nerve blocks for surgical anesthesia and underwent repair utilizing monitored anesthesia care (MAC) with a dexmedetomidine (DEX) infusion.

## Introduction

Hypertrophic cardiomyopathy (HCM) is the most common inherited cardiac malformation that impacts 1 in 500 adults. It follows an autosomal dominant inheritance pattern and has variable genetic expressivity of sarcomere gene mutations resulting in structural abnormalities of the heart [1]. Phenotypically, HCM can present with apical, midventricular, asymmetrical, or concentric hypertrophy of the myocardium, with 70% of HCM cases presenting with hypertrophic obstructive cardiomyopathy (HOCM) [1]. Symptoms typically begin in adolescence, particularly in young athletes, and can manifest as syncope, chest pain, dyspnea, arrhythmia, exercise intolerance, and, most devastating, sudden cardiac death [1].

Devising an anesthetic plan for patients with HOCM poses significant challenges to anesthesiologists and includes careful consideration of the degree of obstruction and heart function, comorbidities, and the type of surgery. Perioperative goals include maintaining adequate volume status, systemic vascular resistance, avoiding tachycardia, reducing contractility, and selecting agents that have minimal or reduced sympathetic effects. While general anesthesia is common, neuraxial and regional anesthesia can also be used cautiously [2-4].

Dexmedetomidine (DEX), a highly selective alpha-2 adrenergic agonist, offers unique direct and indirect cardioprotective stabilization, minimal respiratory depression, anxiolysis, sedation, and analgesia [5,6]. Through its central inhibition of norepinephrine release at the locus coeruleus in the pons, DEX indirectly reduces the sympathetic response during surgery, which is particularly useful for patients with high-risk obstructive and arrhythmia-prone cardiologic conditions such as HOCM [6,7]. DEX shows flexibility in a variety of settings such as patients who are mechanically ventilated in the intensive care unit, under general endotracheal anesthesia, and under monitored anesthesia care (MAC), and can be used as an adjuvant to prolong the analgesic effects of peripheral nerve blocks [5,8-10].

In this case presentation, we highlight the successful and safe use of preoperative peripheral nerve blocks in a patient with HOCM undergoing a right arthroscopic surgical repair under MAC with intravenous DEX.

## Case presentation

The consent for writing the case report was obtained along with the surgical consent. We present the case of a 15-year-old female with a past medical history of HOCM, seasonal asthma, and obesity who came to our institution for elective right knee arthroscopy for patellar instability. She was seen in our institution's preoperative clinic and was cleared by her cardiologist to proceed with surgery. During preop, she denied any concerning symptoms, such as chest pain or dyspnea. However, she is limited to moderate levels of exertion (e.g., jogging a short distance) before becoming short of breath. She was not on any routine cardiac medications like beta blockers or anti-arrhythmic.

In the preoperative area, a point-of-care cardiac ultrasound showed a hypertrophied left ventricle with normal contractile function, as shown in Video 1. A prior echo showed a moderately hypertrophied and hyperdynamic left ventricle (LV) with >65% ejection fraction, mild mitral valve (MV) systolic anterior motion (SAM), and mild MV chordal abnormality causing LV outlet obstruction.

**Video 1:** A preoperative transthoracic echocardiogram (TTE) point-of-care ultrasound (POCUS) in long- and short-axis views reveals a moderately hypertrophied left ventricle LA: left atrium; MV: mitral valve; LV: left ventricle; LVOT: left ventricular outflow tract; RV: right ventricle

After discussing the risks and benefits of general, neuraxial, and regional anesthesia techniques with the patient, family, and surgeon, we ultimately decided to proceed with regional anesthesia for surgical blockade in addition to MAC anesthesia with DEX.

**Figure 1:**
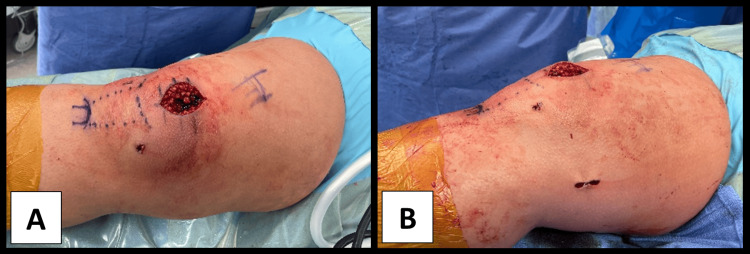
Anterior (A) and medial (B) views of the patient’s right arthroscopic repair prior to closing

The patient underwent ultrasound-guided right femoral nerve block with 20 mL 0.5% ropivacaine and a right infra-inguinal fascia iliaca compartment block with 20 mL 0.2% ropivacaine prior to surgery. The weight-based toxic dose of ropivacaine in milligrams was calculated prior to nerve blockade to ensure patient safety. Once in the operating room, an ice test was performed to confirm appropriate sensory deficit in the femoral nerve distribution. The dexmedetomidine infusion was then initiated at the rate of 0.5 mcg/kg/h. Supplemental oxygen with end-tidal CO_2_ monitoring was applied in addition to American Society of Anesthesiologists (ASA) standard monitors for MAC anesthesia. The final procedure included right knee arthroscopy with loose body removal (5x8 mm) from the medial compartment, lateral release, partial lateral meniscectomy, patellar chondroplasty, medial patellofemoral ligament reconstruction with allograft, and medial quadriceps tendon femoral ligament reconstruction with allograft. Images of the completed surgery prior to closure are shown in Figure 1. The surgical time was 166 minutes, with an estimated blood loss of 30 mL. The intra-operative course was uneventful and the patient was hemodynamically stable with no incidents of arrhythmia or hypotension. The patient experienced no postoperative complications in the post-anesthesia care unit (PACU) and was discharged home on the same day of surgery.

## Discussion

HOCM is characterized by LV hypertrophy, LV outflow tract (LVOT) obstruction, profound diastolic filling impairment, reduced LV compliance, and secondary mitral valve regurgitation [1]. Sub-aortic outlet obstruction occurs due to the collapse of the hypertrophied interventricular septum in the setting of high blood flow velocities, decreased blood volume, increased contractile states, and increased heart rate [1]. Eventually, the hypertrophied tissue outcompetes the oxygen supply, fibrosis ensues, diastolic function worsens, and systolic dysfunction occurs. Due to this, patients with HOCM are at high risk for cardiac arrhythmia and myocardial infarction, requiring extreme caution when caring for these patients in the operating room [11,12].

Developing anesthetic plans for patients with HOCM requires attention to the patient's disease state, comorbidities, and the proposed operation [4]. Hemodynamic goals in HOCM include encouraging preload by increasing intravascular volume, maintaining afterload by increasing systemic vascular resistance to avoid wide LVOT gradients, maintaining a low-normal heart rate, and avoiding increased contractility by blunting the sympathetic response to stimulation and avoiding inotropic pharmacologic medications [1]. Other goals include patient comfort, pain management, and anxiolysis to avoid unwanted sympathetic stimulation [11,13].

For patients with HOCM undergoing orthopedic surgery, techniques such as neuraxial anesthesia and peripheral nerve blockade have been demonstrated to be safe and efficacious [4,14]. Neuraxial anesthesia like epidural or spinal anesthesia was avoided in this case to avoid the risk of a sudden decrease in systemic vascular resistance. The type of regional anesthesia was carefully chosen to provide surgical anesthesia. Since our patient underwent an arthroscopic patellar repair, we chose a femoral nerve block and fascia iliaca compartment block with MAC. Femoral nerve blocks have long been incorporated into orthopedic surgery and are indicated in knee arthroscopy, knee arthroplasty, femur repairs, and cruciate ligament repairs [15]. Zero point five percent (0.5%) ropivacaine was used to achieve surgical anesthesia in the distribution of the femoral nerve along the anterior thigh, anteromedial knee, and medial leg. The fascia iliaca compartment block was additionally performed to provide coverage of the lateral femoral cutaneous nerve and obturator nerve [16].

In addition to providing surgical anesthesia, regional nerve blocks provide better pain control minimizing catecholamine surges, which could cause tachycardia, arrhythmia, and increased myocardial stress. Regional anesthesia also reduces opioid use in the postoperative period and helps reduce opiate-related side effects like postoperative nausea and vomiting (PONV). We used DEX due to its anxiolysis, analgesia, minimal effect on respiratory depression, bradycardic effects, myocardial stability, and reduced incidence of emergence delirium [6,7].

DEX is a highly selective alpha-2 adrenergic agonist that is uniquely advantageous due to its central nervous system and presynaptic actions that reduce peripheral catecholamine release, thus blunting sympathetic stress response during surgery [6]. DEX’s cardioprotective effects include reducing myocardial oxygen demand, systemic vascular resistance, and heart rate, therefore promoting prolonged diastolic filling and myocardial perfusion [17]. Additionally, DEX reduces arrhythmias in high-risk patients, such as HOCM, due to its direct inhibition of cardiac norepinephrine release and its anti-inflammatory and antiapoptotic effects, which lessen ischemic reperfusion injury [6]. In the PACU, DEX is useful for improving perioperative and postoperative pain relief due to its “cooperative sedative” effects, reducing postoperative nausea and vomiting (PONV), agitation, pain, cough, and shivering [5,18-20].

Our patient tolerated the procedure without hemodynamic compromise, had no emergence delirium, and her postoperative course was uncomplicated. In the PACU, she had no postoperative pain, required no postoperative opiates, and had no PONV, which was important given her high risk for PONV.

## Conclusions

In summary, dealing with HOCM presents multiple challenges for anesthesiologists, given the potential for hemodynamic instability and cardiac arrhythmias leading to unfavorable perioperative outcomes. This case underscores the significance of comprehensive preoperative assessments and the skill of customizing anesthetic plans based on individual patient requirements. Anesthesiologists should recognize that regional and neuraxial techniques offer secure alternatives to general anesthesia, especially for patients with advanced diseases or critical conditions. It is crucial for anesthesiologists to be readily available as perioperative consultants for both our surgical colleagues and, more significantly, our patients.
